# The Experiences of First-Time Fathers in Perinatal Services: Present but Invisible

**DOI:** 10.3390/healthcare9020161

**Published:** 2021-02-03

**Authors:** Suzanne Hodgson, Jon Painter, Laura Kilby, Julia Hirst

**Affiliations:** 1 Department of Nursing and Midwifery, Collegiate Crescent, Sheffield Hallam University, Sheffield S10 2BP, UK; J.Painter@shu.ac.uk; 2 Centre for Behavioural Science and Applied Psychology, Sheffield Hallam University, Sheffield S10 2BP, UK; L.Kilby@shu.ac.uk; 3 Department of Psychology, Sociology and Politics, Sheffield Hallam University, Sheffield S10 2BP, UK; J.Hirst@shu.ac.uk

**Keywords:** new fathers, new dads, paternal, service provision, perinatal

## Abstract

Fathers in the UK are becoming more involved in the care of their infants and children. A constructivist grounded theory approach was adopted to explore men’s transition to fatherhood. This paper reports on one of the sub-categories derived from the data. First-time fathers with a child under two were recruited predominantly via social media. Audio-recorded semi-structured interviews were undertaken with an opening question asking men to tell their story of becoming a father. Interviews were transcribed and analysed using constructivist grounded theory methods. This paper reports one core aspect of the research findings which has particular relevance for healthcare professionals. The men in this study were highly appreciative of the care their partner and baby received but consistently reported a lack of father-specific support throughout their journey to fatherhood. This ranged from generally poor communication with healthcare professionals to being ignored and side-lined in maternity settings where they continued to be treated as visitors before, during and after the birth of their baby. Despite similar findings being reported over the last 30 to 40 years and policy directives emphasising the importance of working with fathers, change within healthcare services remains slow. Currently, fathers’ needs are not being adequately met by perinatal services.

## 1. Introduction

Fathers in the UK are increasing the amount of time spent with their children, providing care which has traditionally been seen as a mother’s role [1]. They are now more involved in their children’s care than ever before and have begun to move away from traditional roles of primary breadwinner and disciplinarian to that of a co-parent [2]. This is a relatively recent shift in thinking and although at a micro level, many fathers are embracing the opportunities to care for their infants and children, they have few generational role models on which to base this new style of parenting and frequently express a desire to be different parents to their own fathers [3,4]. Mothers and fathers are now expected to combine a multitude of roles including breadwinning and providing emotional and practical care to their children [5]. At a macro level, there are often significant inhibitors to the adoption of this role for fathers particularly related to workplace requirements. Paternity leave was introduced in the UK in 2003 giving new fathers who were in contracted employment two weeks of leave following the birth of their child, paid at statutory parental leave rates [6]. In 2018, it became possible for mothers and fathers to share parental leave of up to 50 weeks and up to 37 weeks of pay [7]. However, financial implications, the pressure of gendered parenting norms and associated maternal gatekeeping and organisational barriers were all found to affect shared parental leave uptake [8].

Large health care organisations in the UK also appear to operate on a much more traditional basis from a gender role perspective [9]. For many parents, the interactions with healthcare providers perinatally may be the most consistent contact they have with health services in their adult lives outside of serious illnesses. It is now usual, if not an expectation, for men in the UK to be present at the birth of their babies with approximately 98% of men who are co-resident with their partners being present [10]. Current evidence suggests that men do not receive sufficient support or guidance in relation to becoming a father and are often neglected by perinatal health services [11,12]. This is despite explicit guidance in policy such as The Healthy Child Programme in the UK to engage with fathers and strengthen couple relationships throughout pregnancy and the first five years of life [13]. In the UK, midwifery and nursing remain female-dominated professions [14] and research suggests this lack of male representation may present barriers to the development of father-inclusive maternity and well-child services [15]. Authors argue that gender role stereotyping and the use of gendered language lead both professionals and fathers alike to view maternity and well-child services as not being accessible or available to fathers [14,15].

Researchers, and perinatal health practitioners have become increasingly interested in fathers’ experiences and there is a growing evidence base regarding men’s experiences of becoming new fathers, and the effect that fathers’ relationships with their children have on children’s cognitive and emotional development [16,17]. The positive relationships fathers enjoy with their children is shown to impact upon their children’s future relationships and potentially contribute to more stable family lives and more positive role models for future generations of fathers [18].

The impact of poor paternal mental health in the perinatal period has also been documented [19,20,21]. Men with partners with postnatal depression (PND) are more likely to experience PND themselves [22,23]. Paternal PND, just like maternal PND is influenced by a lack of social support, anxiety, pressure and a lack of parenting autonomy [21]. A recent study found that men experience a broad range of challenging emotions in the transition to fatherhood but question the validity of their feelings during this time for fear of taking away professional support or attention from their partners [24], compounding a reticence to share these feelings. This questioning of feelings by fathers may well be influenced by the expectations and attitudes of service providers [25], gendered norms around “manning up” and other such unhelpful sentiments. Men with unrealistic expectations of parenthood and those who lack preparation for the emotional realities of fatherhood have been shown to display higher rates of perinatal distress [26]. Meaningful social support has been shown to be protective of issues such as postnatal depression in both mothers and fathers [27,28]. In a study of both mothers and fathers, high levels of social support antenatally showed less postnatal distress at six weeks [29]. There are numerous studies describing men’s inequitable experiences of antenatal support [12,25,30,31], labour and birth [32,33,34,35,36] and the first year of fatherhood [29,37,38]. Few of these, however, explain why new and expectant fathers feel dissatisfied with their experiences of perinatal service provision. Despite the changing nature of fatherhood, and the aforementioned increase in research focused on fathers, there is still far less contemporary literature regarding their experiences compared to that relating to new mothers. Consequently, the processes involved in men’s transition to fatherhood in the contemporary UK context remains poorly understood.

Whilst previous studies have investigated men’s experiences of various types of health service provision during the perinatal period, the literature is particularly lacking in current qualitative evidence. Therefore, a constructivist grounded theory [39] study was undertaken to explore men’s transition to fatherhood. Constructivist analysis of the transcripts generated three core theoretical categories namely: aspirations for fathering, which divides into subcategories: (i) hoping and preparing, (ii) instigating a transformation, (iii) re-negotiating relationships; tensions in fathering, which divides into subcategories: (i) negotiating the workplace, (ii) navigating gender; (iii) healthcare holds power: being present but invisible; and fluidity of fathering which divides into sub categories: (i) perceiving a role, (ii) role endorsement.

This paper solely reports findings from the sub-category “healthcare holds power: being present but invisible”. The findings contained within this sub-category provide crucial insight regarding the hitherto poorly understood experiences that new fathers have with perinatal healthcare providers in the UK. Hence, they are reported here as standalone findings which will be of particular interest to healthcare professionals. This sub-category includes data from all 12 fathers involved in the study and relates to the interactions these men had with healthcare providers during pregnancy, birth and their first year of fatherhood.

## 2. Methods

This study adopted a constructivist grounded theory methodology (CGTM) [39,40]. At the core of grounded theory methodology (GTM) is an inductive approach which allows a theory to be developed which is grounded in the data collected, most often, but not exclusively, via interviews [41]. CGTM is underpinned by symbolic interactionism focusing on the nature of social interactions [42,43] and is built on the philosophy that individuals construct their own reality based on their experiences of the world and their interactions with others [40]. CGTM is a useful approach for phenomena which are not well understood, or which are under-researched [44]. Men’s experiences of the transition to fatherhood are likely to be influenced by numerous interactions with professionals, community members and their friends and relations. The meaning ascribed to these interactions and the symbolism of language and actions offer important insight into this poorly understood phenomenon.

Following core CGTM principles of constant comparison and theoretical sampling, data collection and analysis were conducted concurrently [45]. An iterative process of line-by-line coding and the formulation of initial categories led to the development of the theoretical categories [46]. CGTM does not seek the development of grand theory, rather it seeks contextually relevant knowledge related to the lives and experiences of those participating in the study. Extant theory is rarely applied to grounded theory studies except to complement the findings of the study itself. As is typical in qualitative research, constructivist grounded theory findings provide insight that is often transferable to other groups or other contexts but generalisability is not the objective of grounded theory research [39].

First-time fathers, initially with a child under the age of one year, were recruited predominantly via social media advertising. Adverts were also placed on a university’s research recruitment website and hosted on some relevant voluntary sector organisation sites. Men responded via email and participant information sheets were emailed back to them, enabling them to make an informed decision about participation. Of the 21 information sheets sent out, twelve men consented and participated over a period of 12 months. Face-to-face, semi-structured, audio-recorded interviews were conducted ranging in length from 40 min to two hours (over one or two sessions). All interviews commenced with the broad question: “can you tell me your story of becoming a father”. Further questions related to experiences of healthcare (reported here), preparation for fatherhood, life change, returning to work, and other topics the fathers themselves identified as being important to them. Audio-recordings were transcribed verbatim by the first author and analysed using constructivist grounded theory methods [39]. Theoretical sampling and constant comparison approaches [47] were used to inform the interview schedule and subsequent inclusion criteria, thus recruitment criteria were expanded to include first-time fathers with a child under the age of two years. Only a handful of previous studies have used constructivist grounded theory methodology to explore the transition to fatherhood in its own right in the UK context. Few studies have had such broad a scope; therefore, this methodological approach was perceived to be appropriate to explain and understand this phenomenon further.

### 2.1. Participants

Twelve new fathers shared their experiences in this study. Six were aged 25–34 and six were 35–44. Eleven participants identified as being of White-British ethnicity with one identifying as White-Other. All were employed for 30 h or more and all had completed formal education. Two completed secondary school, one held a certificate/diploma and four held university degrees. All lived with their partners or spouses; six were married and six were co-habiting. Eleven of the fathers had a baby under one year and one had a child under two years.

### 2.2. Ethics

The study was approved by the university’s research ethics committee (HEC 2015/137 and AM/KW/D&S-311). Informed consent was obtained via signed consent forms which detailed aspects such as audio recording and the grace period for withdrawal from the study. As part of the consent process, men were made aware of how to access support and help, should participation cause negative feelings or emotions, e.g., via their own GP. For some participants, more detailed information was subsequently provided, where they had described traumatic births or suspected postnatal depression, e.g., how to access local birth debriefing counselling. To protect confidentiality, all data were stored in accordance with the university’s GDPR-compliant policies and procedures. Additionally, all names of participants and their families have been changed.

## 3. Results

As outlined above, analysis generated three core theoretical categories, that each divided into sub-categories. The subcategory: healthcare holds power: being present but invisible reports on the participants’ experiences with healthcare providers during their transition to fatherhood. We structure our findings to reflect the order in which our participants described them, i.e., tracing the stages of pregnancy, labour and birth, and finally postnatally.

### 3.1. Ante-Natal Experiences

Little changed physically or emotionally for many of the participants in this study during pregnancy and their perceptions of becoming a father tended to be associated with the onset of labour or the moment of birth. Most suggested that they were somewhat detached from the pregnancy and described it as a somewhat abstract entity:

“I think there’s an immediate sort of responsibility towards your wife and your unborn child… so there’s that aspect of the feelings to provide, to care for, to make sure everything goes ok, but at the same time, as I said before… nothing’s actually changed, for you physically, at that time, so there’s that little bit of disconnect with everything at the same time.”

Alex, p. 8

Antenatal preparation in the form of classes was deemed inadequate, with these fathers feeling they still had little idea of how to care for a new baby:

“So, you went away from that not really knowing how to look after a kid at all, we did a bit of breathing, a bit of massage, how to change a nappy, how to bath it and that were it.”

Neville, p. 2

“It strikes me that a lot of the advice beforehand is tailored towards mothers and perhaps something similar for fathers would be good.”

Mark, p. 16

Two of the new fathers had experienced a miscarriage but neither had received father-focused support or acknowledgement in relation to this. This affected their responses to subsequent pregnancies:

“I mean when we lost [the] first one, we didn’t get any, Marissa got given a sort of pamphlet about counselling, but I didn’t … No, I mean I went outside, and Marissa was really strong, and I broke down in the car park for about sort of twenty minutes… I think after losing [the] first one as well I didn’t pin my hopes on anything.”

Neville, p. 6.

“With the first pregnancy I didn’t really feel that I was that emotionally connected or involved, engaged whatever, whereas the second one, yeah much more so. We were looking out for everything.”

Rob, p. 4

Women frequently feel isolated and alone following pregnancy loss [48]. Despite the perception that there is more acknowledgement of the impact of pregnancy loss on mothers, it may be that there are more similarities than differences in this experience for men and women and improved support is warranted for both parents.

Many of the new fathers wondered why people were not more honest about the realities of becoming a parent and would prefer to have known the truth:

“A more realistic portrayal of what pregnancy is going to be like for their partner, what it’s going to be like for them, of its not all going to be brilliant and all the good stuff they talk about, there is going to hard times, there is going to be a difficult conversation you need to have…, the process of giving birth is not anything like I was prepared for it to be and just kind of more realistic portrayal of things… You see on TV they pass them the baby and it’s all nice and clean, not that it’s got a wonky head or a lump on its head and covered in weird manky stuff…”

Iain, pp. 40–41

Being emotionally and practically prepared for fatherhood reportedly reduced unrealistic expectations and positively impacted upon distress in the transition to fatherhood. Father-inclusive antenatal preparation and an improvement in psychological support for both parents following miscarriage would seem to promote a more positive identity formation for men as they become fathers. It may be that due to social norms related to whose business pregnancy and childbirth is, women are more able to gain informal emotional and preparatory support from friends and family. Men have traditionally found these conversations hard to have with their peers or family with ongoing evidence suggesting that there are prevailing masculine norms which might prevent this [49,50].

### 3.2. Labour and Birth

Participants described this time as one where their autonomy and control was most diminished, where their (and even their partners’) voices were not heard in general, and where they felt particularly side-lined and excluded from decision making.

“Certainly, pregnancy itself you know, the sort of business end, you certainly feel terribly useless, you know, terribly, terribly useless, so…, all I could be was really,…was just there, and to just… offer whatever my wife felt would help, but… you do feel, utterly utterly useless, especially when you see, your loved one in so much pain and there’s just nothing you can do.”

Albert, p. 26

“I’m sat in a little chair in the corner of the hospital thinking, so she’s basically just told me that my partner could die… basically the words were, you might not make it through and there were no, ‘are you ok with this? ‘Do you want any input into this’… I was just like on the back burner a bit, the whole hospital experience weren’t very nice.”

William, pp. 2–3

No being able to stay with their partner and new baby was discussed by every new father in this study and was a cause of significant worry and anxiety for many of them:

“Two things I found the most difficult, was the very first night he was born, I couldn’t stay in the hospital, I had to go back home, and I remember that night as being just awful.”

Frank, p. 2

“I was worried about not being able to help and that was the main thing I was worried about, obviously you know, sad not to be able to spend the first few hours with her and yeah, just excited to go back the next day and to get home and to start our new lives together.”

Albert, p. 16

“I was quite irritated about that as well. I would happily have slept on the floor; I really didn’t care at all.”

Fred, p. 23

Some of the men described traumatic experiences where they feared for the lives of their partner and baby:

“It was scary, possibly the most scary experience that I’ve ever been through especially looking back now, it was so close to losing both of them.”

Mark, pp. 26–27

As evidenced in the quote above, one of the main practical issues encountered was the inability to stay with their partner and new baby for the first night after the birth. When they were allowed to stay, facilities were inadequate and their basic needs were often not met. This included a lack of shower facilities and no access to hot drinks or meals.

### 3.3. Postnatal Experiences

On return home, interviewees described their role as more “husbanding” or partner support than fathering:

“I was just mainly running around, just doing whatever I could to try and make things a bit easier. I don’t think, there was much fathering there as much as there was husbanding. It was very much supportive whatever you know, trying to predict what Louise might need.”

Rob, p. 30

Ensuring their partner’s and baby’s welfare was paramount and most men described this time as a whirlwind especially if they had to return to work after two weeks. A sense of abandoning their partner and baby was described my many on their return to work as well as exhaustion and a preoccupation with their new existence which impacted significantly upon their motivation and productivity at work:

“I remember as well crying on the bus on the first day. I remember falling asleep on the bus in those first few weeks back at work a number of times and luckily waking up in time to get off the bus. So yeah, a combination of just being sleep deprived and really not wanting to be there when I was at work, I felt pretty rotten, definitely for the first few weeks back at work.”

Frank p. 16

Interactions with health professionals postnatally were found to be rare, but when they were present, the felt excluded from conversations:

“Yeah, feel a bit of a spare part sometimes I felt, you know, [the] conversation was just between my wife and the health visitor and… I was just there really, but on the whole, I think the service, that whole system, I think works really well, the amount of visits that we get, but yeah felt a little bit like a spare part I guess.”

Albert, p. 25

Having someone to talk to with similar experiences was highlighted by several of the fathers. This is particularly pertinent in the context of changing friendships post birth and the protective factor of social support for mental health:

“I think yeah just being able to talk to dads that have gone through similar things. Not necessarily within their friendship groups sometimes those conversations we can’t have even amongst friends so to be able to talk to someone outside the group and not known, probably would have helped.”

Mark, p. 16

“I don’t know if possibly I had a bit of depression because it weren’t going right but again no real follow up on that either which was difficult…”

William, p. 23

The current provision of a Monday to Friday 9–5 postnatal well-child service appeared to miss an opportunity to engage with working fathers and relied on third party reporting of dad’s wellbeing, if this was enquired about at all. Unless the men had extended their time at home by using annual leave, they tended to be at work when health visitors visited the home. The men in this study had varying experiences of meeting with practitioners postnatally but those who experienced distress would have potentially benefitted from this interaction. Some of the men suggested alternative means of supporting fathers identifying a significant life change since becoming a father.

## 4. Discussion

### 4.1. Antenatal

Predominantly fathers wanted to be involved, informed and respected in relation to their interactions during pregnancy and required support from both their pregnant partners and professionals [51]. However, in this and previous studies, new and prospective fathers described being excluded from discussions, decision making and services in general and in some studies reported a sense of unworthiness of health service support [52]. This started during the antenatal period with a lack of father-focused information and support to help them mentally prepare for fatherhood. In addition, men seem to be the forgotten partner in relation to support around pregnancy loss which, for one father in this study, contributed significantly to engagement with the subsequent successful pregnancy. Loss in previous pregnancies has also been associated with paternal postnatal depression [53].

It is known that men who received emotional support for themselves during pregnancy had better physical and psychological health [54]. A lack of psychological preparation is closely associated with unrealistic expectations which have been found to predispose both mothers and fathers to postnatal depression [26]. A lack of acknowledgement of fathers, by services, reinforces their feelings of a lack of agency and autonomy in their fathering, compounding any stress or mental health problems which may be manifesting in the transition to fatherhood [11,21,55].

Speaking directly to fathers and asking their opinions during consultations, encourages their involvement and contributions [56]. Acknowledging the presence of fathers in the consulting room and asking for their opinion or thoughts could, it seems, fundamentally change fathers’ perceptions of services.

### 4.2. Labour and Birth

Whilst most of the men in this study showed great appreciation of the time and skills of the practitioners caring for their partners and new babies, there is a common theme of poor communication with, and inclusion of, fathers during labour and birth. Men felt a frequent of loss of control and lack of agency when entering labour and birth with their partners. Whilst appreciating professional expertise they did not always feel engaged in, or sometimes even aware of decision-making processes regarding treatment options, particularly when emergency situations developed.

Fathers are also vulnerable to post-traumatic stress disorder (PTSD) particularly after traumatic births [57], irrespective of whether their partners or practitioners perceived them to be traumatic. Birth-related PTSD can have long term consequences including resentment of the infant, poor quality emotional relationships, and diminished sexual relationships with their female partners [58]. Fathers who have had preterm infants that required a significant stay in intensive care have also shown signs of post-traumatic stress symptoms [59] which could be related to the fear of losing their partner and/or their infant.

The lack of provision for new fathers to stay overnight with their family immediately post birth was raised by most participants in this study. Most men interviewed would happily have stayed in a chair next to their partner’s bed. The potential perception that these dads would add an additional burden to the workload of midwives and nurses on the ward, is unfounded and it is argued that with coordinated and adequate provision they would instead provide support to their new family and hence relieve some of the pressure on an already over-stretched workforce [60]. The negative stereotyping of men in this clinical environment reinforces inequality to both parents. Most men want to be involved in all aspects of their infant’s care and in order to promote true co-parenting, practitioners need to be supported and encouraged to facilitate this.

Not only are men’s ideas of fathering socially constructed but so are those of their health professionals [40]. Society shapes ideas of what it means to be a father and the expectations on fathers which, when set against the UK’s traditionally female-dominated healthcare workforce present significant challenges for the necessary transition from entirely woman-centric to family-focused care. The needs of 21st century fathers may differ greatly from the expectations of those around them due to the slow pace of change in social norms [61]. A number of NHS trusts around the UK are now inviting fathers to stay with their new family on the first night post birth which is a welcome step forward in making dads feel part of service provision.

### 4.3. Postnatal

The men in this study all described current paternity leave provision as inadequate, finding the first two weeks of parenthood to be overwhelming, exhausting and a huge learning curve. Their lives had changed beyond recognition and they felt that having more time at home to adjust to their new roles would have been incredibly helpful, often dreading returning to work [20]. The husbanding/partnering which these fathers described in the first few days and weeks post birth is mirrored in other studies which suggest that men perceive their role to be less involved with their infant than the mother during this time [62,63]. This perspective is also reinforced by traditional gendered norms around the roles mothers and fathers undertake with infants [64,65] and could be excluding men from bonding opportunities. Bathing and changing nappies are bonding opportunities for both mothers and fathers [66].

Including new fathers more during health visitor consultations and ‘well child’ checks would support their transition to fatherhood. This was often not possible due to the timings of visits and the norm that most men were returning to work after two weeks of paternity leave. More creative ways of engaging both resident and non-resident fathers could support their fathering and provide them with a sense of being included in decision making and in being able to share their concerns and have questions answered. Asking “how are you, dad?” [67] indicates to the father that they are also involved and affected by the process of becoming a parent. There is a growing momentum from advocates of paternal wellbeing to have new fathers included in screening for postnatal depression and anxiety as standard which is currently not usual practice [19,50]. However, identifying fathers who are experiencing perinatal distress, will require that current service provision develops and adapts to meet their unique needs [68].

Overall, this study concurs with much of the existing evidence which suggests that fathers’ needs are not being adequately met by perinatal services [11,69,70,71]. Despite similar findings being reported since research in this area began (as far back as the early 1980s) and despite working with whole families including fathers being a key emphasis of NHS policy since 2004 [18], change within services has been slow. Shifts in the nature of a father’s role in their children’s life from their own and others’ perspectives also requires father-inclusive practices in maternity and well-child services. A sense of being valued and supported could impact significantly upon men‘s involvement with their infants and engagement with co-parenting. The findings of the broader grounded theory study from which this article is drawn, indicate that there may be more similarities than differences in the experiences of men and women in the transition to parenthood and that systemic differences in the way new mothers and new fathers are treated and acknowledged during this time may account for inequalities in service provision faced by men. Unconscious bias, conveyance of personal gendered norms and stereotyping contribute to these experiences [72]. This also impacts upon women’s experiences in healthcare provision, opportunities in the workplace and their experience in broader social contexts and ultimately impacts upon family life overall.

### 4.4. Strengths and Limitations

This study’s strengths stem from its constructivist grounded theory approach which not only gave a voice to fathers trying to navigate services during the perinatal period, but also to understand the underpinning reasons for their views and experiences. The results chime with other studies and it is likely that their experiences mirror those of other men going through the transition to fatherhood. Any study which offers men a voice during this tumultuous period contributes to an important, but relatively scant, body of knowledge and, in this case, reinforces the need for perinatal services to evolve in order to better accommodate new fathers. Framed through a social constructivist lens [39,44], this grounded theory study acknowledges that men’s perceptions of fatherhood will be formulated and based on their interactions and experiences, such as their individual biographies of being fathered, their interactions with other fathers, mothers, service providers, the media and wider society throughout their life course.

The main limitation of this study stems from its qualitative design and scale which preclude generalising its findings to all new fathers. This study presents the experiences of a limited number of men and does not necessarily reflect the experiences of all men as they navigate perinatal services. This group of fathers was self-selecting and was not ethnically or culturally diverse, requiring further study into cultural differences in the transition to fatherhood. Further research should also include gay fathers, social father’s, young fathers and include fathers from more diverse backgrounds both ethnically and socially.

## 5. Conclusions and Implications for Practice

The men in this study all reported a high appreciation for the care that their partner and baby received but consistently highlighted a lack of father-specific support at all stages in their journey to, and through early fatherhood. Identified here are a lack of father-inclusive antenatal education and poor communication with professionals in addition to experiences of being ignored and physically excluded from maternity wards where they continue to be treated as visitors.

This paper clearly demonstrates a need for more father-friendly perinatal health services. Tangible actions generated from these twelve fathers include:

- Father-inclusive planning, decision making and preparation for parenthood.
- Provision for new fathers to stay with their partner and infant immediately post birth.
- Improved facilities for men on maternity wards to promote inclusivity.
- Awareness raising in the perinatal workforce to acknowledge and promote the evolving role of fathers in contemporary society.

In addition to the above points of practice, further research is required to improve our understanding of the experiences of birth partners and same-sex partners in perinatal health services.

Positive experiences do run through these data but often the negative experiences outweigh these. These men’s experiences mirror much of the previous research in this area and so questions need to be asked as to why practice remains slow to change. The health sector must address the needs of fathers by providing more appropriate, father-focused support for men in the perinatal period. Future studies need to explore this issue further to identify the barriers to change in perinatal service provision.

## Data Availability

Data available on request due to restrictions e.g., privacy or ethical. The data presented in this study are available on request from the corresponding author. The data are not publicly available due to compliance on the basis of the participants’ consent.
